# Is Bayh-Dole Good for Developing Countries? Lessons from the US Experience

**DOI:** 10.1371/journal.pbio.0060262

**Published:** 2008-10-28

**Authors:** Anthony D So, Bhaven N Sampat, Arti K Rai, Robert Cook-Deegan, Jerome H Reichman, Robert Weissman, Amy Kapczynski

## Abstract

The US Bayh-Dole Act encourages university patenting of inventions arising from publicly funded research. Lessons from three decades of US experience serve as a cautionary tale for those countries that may choose to emulate Bayh-Dole.

Recently, countries from China and Brazil to Malaysia and South Africa have passed laws promoting the patenting of publicly funded research [1,2], and a similar proposal is under legislative consideration in India [3]. These initiatives are modeled in part on the United States Bayh-Dole Act of 1980 [4]. Bayh-Dole (BD) encouraged American universities to acquire patents on inventions resulting from government-funded research and to issue exclusive licenses to private firms [5,6], on the assumption that exclusive licensing creates incentives to commercialize these inventions. A broader hope of BD, and the initiatives emulating it, was that patenting and licensing of public sector research would spur science-based economic growth as well as national competitiveness [6,7]. And while it was not an explicit goal of BD, some of the emulation initiatives also aim to generate revenues for public sector research institutions [8].

We believe government-supported research should be managed in the public interest. We also believe that some of the claims favoring BD-type initiatives overstate the Act's contributions to growth in US innovation. Important concerns and safeguards—learned from nearly 30 years of experience in the US—have been largely overlooked. Furthermore, both patent law and science have changed considerably since BD was adopted in 1980 [9,10]. Other countries seeking to emulate that legislation need to consider this new context.

## Overstating Claims

On a positive note, the BD Act required different agencies that funded US research and development to adopt more consistent policies about ownership of patents arising from federal funding [5]. One of BD's intended virtues involved transferring default patent ownership from government to parties with stronger incentives to license inventions. BD assigned ownership to institutions, such as universities, nonprofits, and small businesses, although it could just as easily have opted for individual grant and contract recipients.

Nevertheless, many advocates of adopting similar initiatives in other countries overstate the impact of BD in the US. Proponents note *The Economist's* 2002 claim that the Act was “[p]ossibly the most inspired piece of legislation to be enacted in America over the past half-century” [11]. They also cite data (originally used by US proponents of the Act) on the low licensing rates for the 28,000 patents owned by the US government before BD to imply that the pre-BD legal regime was not conducive to commercialization [12]. But as Eisenberg [5] has argued, that figure is misleading because the sample largely comprised patents (funded by the Department of Defense) to which firms had already declined the option of acquiring exclusive title. Moreover, these figures are of questionable relevance to debates about public sector research institutions, because most of the patents in question were based on government-funded research conducted by firms, not universities or government labs [13]. Finally, and most importantly, the narrow focus on licensing of patented inventions ignores the fact that most of the economic contributions of public sector research institutions have historically occurred without patents—through dissemination of knowledge, discoveries, and technologies by means of journal publications, presentations at conferences, and training of students [6,14,15].

Throughout the 20th century, American universities were the nation's most powerful vehicles for the diffusion of basic and applied research results [16], which were generally made available in the public domain, where industry and other public sector researchers could use them. These activities were central to the rise of American technological success broadly and to the growth of knowledge-based industries, such as biotechnology and information technology, in particular.

Public sector research institutions also relied on generous public funding for academic research—from a highly diverse group of federal funding agencies—which grew dramatically after the Second World War, and on the availability of venture capital to foster the development of early-stage ideas [6]. These and other unique features of the US research and development system explain much more about innovation in the US after BD than the rules about patenting that BD addressed.

In the pre-BD era, discoveries emanating from public research were often commercialized without patents, although academic institutions occasionally patented and licensed some of their publicly funded inventions well before BD, and these practices became increasingly common in the 1970s [17]. Since the passage of the Act in 1980, US academic patenting, licensing, and associated revenues have steadily increased. BD accelerated this growth by clarifying ownership rules, by making these activities bureaucratically easier to administer, and by changing norms toward patenting and licensing at universities [6]. As a result, researchers vested with key patents sometimes took advantage of exclusive licenses to start spin-off biotechnology companies. These trends, together with anecdotal accounts of “successful” commercialization, constitute the primary evidence used to support emulating BD in other countries. However, it is a mistake to interpret evidence that patents and licenses have increased as evidence that technology transfer or commercialization of university technology has increased because of BD.

Although universities can and do patent much more in the post-BD era than they did previously, neither overall trends in post-BD patenting and licensing nor individual case studies of commercialized technologies show that BD facilitated technology transfer and commercialization. Empirical research suggests that among the few academic patents and licenses that resulted in commercial products, a significant share (including some of the most prominent revenue generators) could have been effectively transferred by being placed in the public domain or licensed nonexclusively [6,18].

Another motivation for BD-type legislation is to generate licensing revenues for public sector research institutions. In the US, patents are indeed a source of revenues for some universities, but aggregate revenues are small. In 2006, US universities, hospitals, and research institutions derived US$1.85 billion from technology licensing compared to US$43.58 billion from federal, state, and industry funders that same year [19], which accounts for less than 5% of total academic research dollars. Moreover, revenues were highly concentrated at a few successful universities that patented “blockbuster” inventions [20].

A recent econometric analysis using data on academic licensing revenues from 1998 to 2002 suggests that, after subtracting the costs of patent management, net revenues earned by US universities from patent licensing were “on average, quite modest” nearly three decades after BD took effect. This study concludes that “universities should form a more realistic perspective of the possible economic returns from patenting and licensing activities” [21]. Similarly, the head of the technology licensing office at MIT (and former President of the Association of University Technology Managers) notes that “the direct economic impact of technology licensing on the universities themselves has been relatively small (a surprise to many who believed that royalties could compensate for declining federal support of research)… [M]ost university licensing offices barely break even” [22].

It is thus misleading to use data about the growth of academic patents, licenses, and licensing revenues as evidence that BD facilitated commercialization in the US. And it is little more than a leap of faith to conclude that similar legislation would automatically promote commercialization and technology transfer in other, very different, socioeconomic contexts.

## Sources of Concern

What have we learned from the US experience with BD? Because the Act gives recipients of government research funds almost complete discretion to choose what research to patent, universities can patent not only those inventions that firms would fail to commercialize or use without exclusive rights, but also upstream research tools and platforms that do not need patent protection and exclusive licensing to be adopted by industry [6,9,10].

For example, while the patented technologies underlying recombinant DNA were fundamentally important for biotechnology and generated ample revenues for Stanford, the University of California, Columbia University, and City of Hope Medical Center [6], the patenting and licensing of these research platforms and technologies were not necessary for commercialization. Both the Cohen-Boyer patents for recombinant DNA and the Axel patents on cotransformation were rapidly adopted by industry even though neither invention came with the BD “carrot” of an exclusive right. The Cohen-Boyer patents reportedly contributed to 2,442 new products and US$35 billion in sales. Its licensing revenues to Stanford University and the University of California San Francisco were US$255 million [23]. With 34 firms licensing the technology, the Axel patents earned US$790 million in royalties for Columbia University over the patent period (Colaianni and Cook-Deegan, unpublished data). While the patenting and licensing of these inventions clearly enriched the universities involved, there is no reason to believe that nonexclusive licensing (as opposed to simple dedication to the public domain) deterred commercialization of the invention(s). In fact, Columbia University justified efforts to extend the life of its Axel patents not because such extension would improve commercialization, but rather because it protected royalty income that would be channeled back into its educational and research mission.

While BD gave those conducting publicly funded research the discretion to patent fundamental technologies, changes in US patent law since 1980 provided the means, by expanding eligibility standards to include basic research and research tools. These trends have been notable in the biotechnology and information technology sectors [24,25]. A widely watched, recent consequence of this shift involves the suite of University of Wisconsin patents on embryonic stem cell lines [26–28]. Biotechnology firms eager to do research on stem cells have complained about the excessive licensing fees that Wisconsin charges (as well as about “reach through” provisions that call for royalties on any product developed from research on embryonic stem cells, and impose restrictions on use) [29]. Rather than promote commercialization, these patents on basic research platforms constitute a veritable tax on commercialization [30]. Nor were these efforts to tax future innovation unprecedented, as the example of recombinant DNA shows. The Wisconsin Alumni Research Foundation's extension of licensing terms to academic research institutions [31] and its imposition of restrictions on use became especially controversial because these measures went beyond the Cohen-Boyer precedent. The manager of recombinant DNA licensing at Stanford quipped, “[W]hether we licensed it or not, commercialization of recombinant DNA was going forward…a nonexclusive licensing program, at its heart, is really a tax…But it's always nice to say ‘technology transfer’” [32].

The broad discretion given to publicly funded research institutions to patent upstream research raises concern about patent thickets, where numerous patents on a product lead to bargaining breakdowns and can blunt incentives for downstream research and development (R&D) [33,34]. Barriers to bundling intellectual property necessary for R&D become higher in frontier interdisciplinary research areas, such as synthetic biology, microarrays, and nanobiotechnology, because they draw upon multiple fields, some of which may be likelier than others to form thickets over time [9,10,32,35]. Although there is some evidence that biotechnology and pharmaceutical firms may be able to avoid thickets through secret infringement or by “off-shoring” research to countries with fewer patent restrictions [36], secret infringement and the transfer of R&D to other countries are hardly tactics that government policy should encourage.

The problems that BD has raised for the biopharmaceutical industry are dwarfed by the problems it has raised for information technology. Universities may too often take a “one size fits all” approach to patenting research results, notwithstanding the evidence that patents and exclusive licensing play a much more limited role in the development of information technology than they do in the pharmaceutical sector [37]. In testimony to the US Congress, a prominent information technology firm complained that aggressive university patenting impeded both product development and university–industry collaboration, which encouraged companies to find other university partners, often outside the US [38]. Expressing similar concerns in a proposal to explore alternatives to the BD model, officials from the Ewing Marion Kauffman Foundation (the leading US foundation supporting entrepreneurship research) recently argued that “Technology Transfer Offices (TTOs) were envisioned as gateways to facilitate the flow of innovation but have instead become gatekeepers that in many cases constrain the flow of inventions and frustrate faculty, entrepreneurs, and industry” [39].

These problems have not escaped the attention of funding agencies, most notably the US National Institutes of Health (NIH), which has issued guidelines stating that patents should be sought, and exclusive licenses should be restricted, only when they are necessary for purposes of commercialization [40,41]. Beyond such hortatory guidelines, however, US funding agencies retain very limited authority to guide the patenting and licensing practices of publicly funded research institutions. Under BD, agencies can declare particular areas off-limits to patenting only when they find “exceptional circumstances.” Moreover, they must present this decision to the Department of Commerce, the primary administrator of BD. The “exceptional circumstances” authority has only rarely been used [30]. However, when exclusive licensing demonstrably impeded commercialization, the funding agencies did not intervene by exercising their authority to mandate additional licensing. Their reluctance to take such action stems in part from the realization that, under the BD regime as enacted, any mandate could immediately be challenged (and its effect stayed) pending the outcome of protracted litigation [30].

Some of the top US universities have themselves begun to recognize the difficulties that overly aggressive proprietary behavior can engender, as demonstrated by their March 2007 declaration highlighting “Nine Points to Consider in Licensing University Technology” [42]. How this declaration will affect university behavior is difficult to predict. Moreover, the “Nine Points” declaration focuses almost entirely on licensing and fails to address how universities should determine whether patents are necessary for commercialization in the first instance.

BD has also led to downstream concerns. The BD framework makes minimal reciprocal demands from licensees of government-funded technologies, and neither universities nor government agencies have sought to include requirements that products derived from these inventions be sold to consumers on reasonable terms [43]. Nor do funders require either disclosure of follow-on investments, so that prices might reflect the private contribution to development or the avoidance of abusive or anticompetitive marketing practices [43–47].

Some have raised concerns that the Act contributed to a change in academic norms regarding open, swift, and disinterested scientific exchange [48,49]. For example, in a survey to which 210 life science companies responded, a third of the companies reported disputes with their academic collaborators over intellectual property, and 30% noted that conflicts of interest had emerged when university researchers became involved with another company [50]. Nearly 60% of agreements between academic institutions and life science companies required that university investigators keep information confidential for more than six months—considerably longer than the 30 to 60 days that NIH considered reasonable—for the purpose of filing a patent [50]. Similarly, in a survey of life science faculties at universities receiving the most NIH funding, nearly a third of the respondents receiving a research-related gift (e.g., biomaterials, discretionary funds, research equipment, trips to meetings, or support for students) reported that the corporate donor wanted pre-publication review of any research articles generated from the gift; and 19% reported that the companies expected ownership of all patentable results from the funded research [51].

Although the surveys discussed above were conducted in the mid to early 1990s, their findings appear robust over time. In a more recent survey of university geneticists and life scientists, one in four reported the need to honor the requirements of an industrial sponsor as one of the reasons for denying requests for post-publication information, data, or materials [52]. This finding is also corroborated by a survey of US medical school faculty. In these settings, researchers most likely to report being denied research results or biomaterials by others were “those who have withheld research results from others” or who had patented or licensed their own inventions [53]. So the practices of patenting and licensing clearly encumber the openness of scientific exchange in universities.

## Instituting Safeguards

Countries seeking to enhance the contributions of universities and public sector laboratories to social and economic development have numerous policy options. Many of these policies do not involve intellectual property rights at all, but rather look to provide funds for basic and applied research, subsidize scientific and engineering education, strengthen firms' ability to assimilate university research, and invest in extension, experimentation, and diffusion activities [39,54,55]. But even policies focused on intellectual property management need not presume that patenting and exclusive licensing are the best options. For example, they may instead focus on placing by default or by strategy government-funded inventions into the public domain, creating a scientific commons, enabling collective management of intellectual property, or fostering open-source innovation [56–60]. Where greater commercial incentives seem necessary, the benefits of nonexclusive licensing should always be weighed against the social cost of exclusive licenses.

The appropriate array of policies will vary from country to country: there is no “one size fits all” solution. Based on our review above, we believe it is doubtful that the benefits of legislation closely modeled on BD would outweigh their costs in developing counties. For those countries that nonetheless decide to implement similar laws, the US experience suggests the crucial importance, at a minimum, of considering a variety of safeguards (see Box 1).

**Box 1: Safeguards Serving the Public Interest**

Governments adopting laws styled after the US BD Act should be vigilant to ensure that the public's interests are served. In commercializing publicly funded research, a number of safeguards on patenting and licensing practices should be built into any law or its regulatory implementation.

**No Exclusive Licensing Unless Necessary for Commercialization**

Any BD-style legislation should be founded on the principle that publicly funded research should not be exclusively licensed unless it is clear that doing so is necessary to promote the commercialization of that research. Public sector institutions should not, for example, exclusively license research tools that were developed with public funding if those tools can instead be used off the shelf by others. Where exclusive licenses are not required for commercialization, one may ask whether universities and public sector labs should be patenting research at all. Will encouragement of patenting and nonexclusive licensing, as in the Cohen-Boyer model discussed above, help or hurt researchers, firms, and the public in developing countries? Even nonexclusive licenses will tax downstream users, although presumably with lower rents and transaction costs and more procompetitive effects. As suggested above, revenues from licensing academic inventions are likely to be minuscule for most institutions, and aggressive university patenting can have other deleterious effects. A robust research exemption can ward off some of the problems potentially associated with restrictive licensing of upstream inventions [62].

**Transparency**

The legislation should ensure transparency in the patenting and licensing of publicly funded research. Public accountability should follow public funding. Institutions that engage in patenting and licensing should be required to report or make public all information that is necessary to determine whether they are reasonably serving the public interest. Such information may include the number of patents and licenses obtained, the funds expended on patenting and licensing activities, licensing revenues, and the key terms (e.g., exclusive or nonexclusive, humanitarian access, research exemption, definition of market segmentation or field of use, performance milestones, and march-in rights) of licenses. The lack of a transparency mandate is a key flaw of the BD Act that should not be replicated.

**Government Authority To Issue Additional Licenses**

Where licensing arrangements for publicly funded research do not achieve public interest objectives, governmental authorities must have power to override such licenses and to grant licenses to additional or alternative parties [9,10,43]. In the US, this authority is formally embodied in the government's “march-in” rights under BD, but this power has never been exercised. Petitions to invoke it have been made a few times [46,47,63,64], but they have never been granted, and because of the administrative disincentives built into BD, this power is unlikely ever to be used [30]. To avoid this result, legislatures must develop standards to ensure that march-in rights or comparable authority will be exercised when public interest objectives are not otherwise attained.

In evaluating licensing options, those receiving government research funding could also be required to consider the option of licensing patented inventions to a “technology trust,” that is, a commons that would ensure designated inventions remained available to all interested parties on predetermined terms. Such a commons could enable the pooling of socially useful bundles of technology, particularly research tools and health technologies for neglected or rare diseases. Governments might also consider reducing or waiving patent application and maintenance fees for such inventions when they are made broadly available for research and humanitarian application, without royalty, for a specific geographical area or field of use.

**Government Use Rights**

The government should retain an automatic right to use any invention arising from its funding. Under BD, the US government has an automatic “nonexclusive, nontransferable, irrevocable, paid-up license” [65] to use any invention developed with government funds. Typically, however, it does not invoke such a license and often pays monopoly prices for products that it funded. The US experience shows the importance both of establishing that the government should be provided with an automatic license in products resulting from its funding and of elaborating standards to ensure such licenses are actually exercised in appropriate circumstances.

From a broader perspective, governments retain the right to use any invention, whether or not it arises from public funding, under international law [66]. Governments may choose to use patented inventions to promote public health [67], national security [66], or comparable objectives, while public-interest compulsory licenses may sometimes be granted to avoid abusive licensing practices or to ensure access to patented research products on reasonable terms and conditions [43,66]. Where publicly funded grantees fail to commercialize a technology appropriately or to foster its availability, the trigger for government use—under any enabling provision adopted in domestic law—must work better than the march-in right has under BD.

**Access to End Products**

Besides promoting commercialization, the government must ensure consumer access to end products. The public is entitled to expect that the inventions it paid for will be priced fairly. The US experience shows that a BD system that lacks mandatory rules concerning the affordability of end products will not deliver on this reasonable expectation [43–47]. As a condition of receiving a license to a government-funded invention, parties should be required to ensure that end products are made available to the public on reasonable terms and conditions. What constitutes “reasonable” will vary by national context, but it is important to ensure that the term is defined with enough precision to be enforceable.

Licenses to government-funded inventions should presumptively include access-oriented licensing provisions that address humanitarian needs in other countries [68]. One such provision is an open license for production and sale of end products in (or to) developing countries in exchange for a fair royalty [69]. At the very least, when inventions have foreseeable applications in resource-poor regions, a plan for access in those regions should be explicitly incorporated into technology licensing.

## Conclusion

While policies supporting technological innovation and diffusion contribute to economic growth and development, the appropriate sets of policies to harness public sector R&D are highly context-specific. Much depends on factors such as the level of publicly funded research, the focus of such research on basic versus applied science, the capabilities of industry partners, and the nature of university–industry linkages [54,55].

Recognizing these difficulties, reasonable minds may disagree about the likely impact of BD-type legislation elsewhere. Nevertheless, the present impetus for BD-type legislation in developing countries is fueled by overstated and misleading claims about the economic impact of the Act in the US, which may lead developing countries to expect far more than they are likely to receive. Moreover, political capital expended on rules of patent ownership may detract from more important policies to support science and technology, especially the need for public funding of research. Given the low level of public funding for research in many developing countries, for example, the focus on royalty returns at the expense of public goods may be misplaced [61]. Furthermore, it is unclear whether any of the positive impacts of BD in the US would arise in developing countries following similar legislation, absent the multiagency federal pluralism, the practically oriented universities, and other features of the US research system discussed above.

In any event, both the patent laws and patterns of scientific collaboration have changed substantially since BD was passed in 1980. To the extent that legislation governing the patenting and licensing of public sector research is needed in developing countries at all, it should reflect this new context rather than blindly importing a US model that is 30 years old.

## Abbreviations

BD: Bayh-Dole
NIH: National Institutes of Health
R&D: research and development
